# Cultural adaptation and validation of the Attribution Questionnaire for stigma towards disability pension applicants for use among psychiatrists and general practitioners in Sweden

**DOI:** 10.1186/s40359-021-00523-8

**Published:** 2021-02-08

**Authors:** Ashley McAllister, Bo Burström, Patrick Corrigan

**Affiliations:** 1 Department of Global Public Health, Karolinska Institutet, Stockholm, Sweden; 2 Melbourne School of Population and Global Health, The University of Melbourne, Melbourne, Australia; 3 Department of Psychology, Illinois Institute of Technology, Chicago, USA

**Keywords:** Cross-cultural adaptation, Expert committee, Mental illness, Validation, Stigma, Structural equation modelling

## Abstract

**Background:**

This study aimed to culturally translate the Attribution Questionnaire (AQ) to the Swedish language and examine the reliability and validity of the new Swedish version to measure stigma towards disability pension applicants in the Swedish context among psychiatrists and general practitioners.

**Methods:**

The AQ was translated from the original English version into Swedish using the recommended guidelines for cultural translation of questionnaires. Steps included forward/back-translation, use of expert committee and pretesting. Cronbach’s alpha was used to determine internal consistency and structural equation modelling (SEM) was used to test the responsibility model of stigma compared to the original English version.

**Results:**

1,414 physicians completed the questionnaire (23.6%). Cultural translation resulted in many modifications to the original questionnaire to increase the external validity. Internal reliability of the AQ Swedish version (AQ-S) was 0.733 and is considered acceptable. Pity and Segregation-coercion sub-scales showed limited consistency. SEM findings show that the responsibility model of stigma is an acceptable fit for the Swedish setting.

**Conclusion:**

Findings show that the AQ-S is comparable to the other versions of the AQ and is a reliable measure to assess and monitor stigma among physicians in the Swedish setting. Our study shows that cultural translation does not significantly impact the validity of the questionnaire.

## Background

Reducing stigma is a key priority for the Swedish Government [1]. Evidence shows that mental illness-related stigma among health care providers is a barrier to access treatment recovery [2] and contributes to the burden of disease [3]. However, few stigma measures exist that are culturally appropriate for the Swedish context. In this study, we aim to culturally translate the widely validated Attribution Questionnaire [4] to measure the attitudes of Swedish physicians, particularly related to disability pension applicants.

### A brief overview of stigma

In this paper, stigma is defined as a social response to any feature that deviates from the ‘norm’ and leads to others discrediting the individual with that feature [5, 6]. We can break stigma down into three social responses—stereotypes (e.g. beliefs), prejudice (e.g. negative attitudes) and discrimination (e.g. unfair treatment of others based on stereotypes or beliefs). Researchers have idenifed several different types of stigmas including public, self, perceived, vicarious, and structural; two have dominated large scale studies: public and self-stigma the former was the target of this study [7, 8]. Negative attitudes and discrimination directed towards individuals with mental health problems as a result of their condition is public stigma. Self-stigma occurs when the person with a mental illness internalises and applies the negative attitudes and discrimination to themselves. In this paper, we focus on the former, public stigma, in the context of physicians. We know that like the general population, physicians also hold negative attitudes [2, 3] but to the best of our knowledge, physicians’ attitudes towards people with mental illness has not been measured in the Swedish context particularly related to disability pension.

### Main ways to combat public stigma

Research suggests two main ways to change the stigma of many illnesses [8, 9]. Educational approaches incorporate factual information to challenge myths about mental illness; e.g., contrary to the myth of people with mental illness being dangerous, epidemiological research shows prevalence is very low. Contact challenges stigma when the general public has interactions with people with lived experience of mental illness and recovery. Meta-analyses suggest that contact approaches have a significantly better impact than educational approaches [10, 11].

### Attribution theory and stigma

**Fig. 1:**
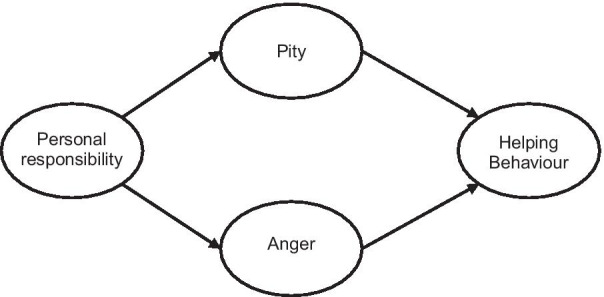
Responsibility model of stigma [4]. *Source*: Adapted from [4]

Attribution theory has been used to explain stigma related to mental illness [4]. Weiner [12] argues that when presented with an event or situation, people try to determine who is responsible. If individuals are not held responsible (external attribution), then others are more likely to engage in helping behaviour. In contrast, if an individual is held responsible (internal attribution), then others are more likely to engage in punishing behaviour. Corrigan and Markowitz [4] drew on attribution theory to develop a conceptual model explaining public stigma related to mental illnesses. The widely tested and validated model (see Fig. 1) suggests that judgements about personal responsibility are predictors of behaviours. Previous studies show a correlation between such attitudes and subsequent behaviours [13]. We chose the Attribution Questionnaire because the questionnaire has been validated in many countries and across different population sub-groups, including physicians [14–19]. We chose this model because this study is part of a larger study measuring physicians’ attitudes toward disability pension—government payments to those unable to work as a result of their disability—applicants with mental illness. In the broader study, we hypothesize that attribution of personal responsibility is a central tenant to the disability pension process and could influence physicians’ behaviours in this process.

### Cross-cultural adaptation of the Attribution Questionnaire

The Attribution Questionnaire [4] (see Additional file 1: Table 1 for original questions) is an American questionnaire that we wanted to culturally translate in the Swedish context. The original Attribution Questionnaire has nine stigmatizing constructs about people with mental illness. In this study, we focus on four of those nine constructs. Responsibility (blame): people have control over and are responsible for their mental illness and related symptoms. Anger: irritated or annoyed because the people are to blame for their mental illness. Pity: sympathy because people are overcome by their illness. Help: the provision of assistance to people with mental illness. The questionnaire uses a hypothetical vignette patient (Harry) who has schizophrenia [4]. Participants rate how much they agree with each statement made about the patient on a Likert scale from 1 (not at all) to 9 (very much) [9]. Yang et al. [20] argue that although stigma appears to be a universal phenomenon, how stigma manifests could be culturally specific. Building on this argument, items to measure stigma should not be one-size-fits-all. The Attribution Questionnaire has been tested and validated in other settings, for example, Italy [14, 18, 19], Spain [15] and Turkey [17]. However, to the best of our knowledge, only the Turkish study adapted the Attribution Questionnaire both for language *and* culture [17]. In fact, in two of the translated versions, the authors note that a significant limitation of the translated version was that it excluded cultural variability. Cross-cultural adaptation of questionnaires goes beyond translating items from one language to another. The purpose also ensures that the translated text is culturally meaningful for use the target population in the new setting [21]. Adaptation is not only respectful to the culture; research shows that it is also essential to maintain the content validity of the instrument [22, 23]. Cultural adaptation has become popular in the health status questionnaires [21] (e.g. the International Quality of Life Assessment), and we want to apply it to stigma measurements.

Overall, we aim to describe the process used to culturally adapt and validate the Attribution Questionnaire in the Swedish context. More specifically, the research questions were:

1. What aspects (if any) of the modified version of the Attribution Questionnaire need modification for the Swedish context?
2. How does cultural adaptation impact the validity of the questionnaire?

## Methods

The translation and cross-cultural adaptation of the Attribution Questionnaire were performed according to the cultural translation guidelines indicated in the scientific literature [21, 22, 24] that include forward translation, back translation, consultation with an expert panel, pretesting and final consensus. We aimed to maintain the integrity of the Attribution Questionnaire while adapting the items to the Swedish cultural equivalents. The following describes how those steps were applied in this study:

### Expert committee

The expert committee comprised representatives from the Swedish Social Insurance Agency, psychiatrists, psychologists, general practitioners (GPs) and researchers. All members had some mental health or medical background. The committee discussed the different versions of the translations, content of the vignettes and suitability of the wording for the Swedish context. To achieve equivalence between the source and target documents, the committee was asked to make decisions on four areas of cultural translation (see Table 1). For this study, between March 2017 and May 2018, we met two times and corresponded via email. We focused our cultural translation using the expert committee rather than backwards translation. The committee was bilingual in Swedish and English. However, we did also professionally back translate the documents in the process.

**Table 1:** Socio-demographic characteristics of the sample (n = 1,414)

Variable	Frequency (n)	Percentage (%)
Type of physician		
Psychiatrist	443	31
GP	971	69
Gender		
Male	722	48.9
Female	691	51.1
Age		
25–34 years	135	9.6
35–44 years	303	21.4
45–54 years	278	19.7
54–64 years	389	27.5
65–74 years	277	19.6
75+	32	2.3
Type of clinic		
Public	851	60.2
Public–private	66	4.7
Private	345	24.4
Other	152	10.8

### Cultural translation

We used one of the items in the helping subscale to illustrate how we applied the cultural translation steps. One item asks whether the respondent would share a carpool with the vignette patient each day. First, a professional translator translated the question from English to Swedish. Then, a bilingual member of the research team checked the translation. Both the English and Swedish versions were sent to the expert committee for review. The committee discussed whether translation was accurate from a semantic perspective i.e. does the Swedish version ask what the English version intended? But also, whether the questions met idiomatic—colloquialisms, or idioms that are challenging to translate, experiential—questions that try capturing everyday experiences and life in the setting and conceptual equivalence—words that hold different conceptual meanings between cultures [21].

The lead author presented the different types of equivalences to the committee and provided examples. The committee then discussed each item to determine if any further modifications were needed. Consensus among the committee showed that carpool is not a experience used in Sweden and suggested that this might be more an American experience. In Sweden, you may give a person a ride but it usually is more casual rather than a regular occurrence and needed to be convenient for the driver. At this point, we changed the carpool to give a ride in your car. We then did cognitive testing^1^ [25] using 3 experts—two medical doctors who are also public health researchers and one psychiatric epidemiologist. All agreed this question was still problematic and suggested some changes. At the next expert committee meeting, discussions continued that this item was problematic and still not suited to the Swedish context. Consensus led to changing the questions from “I would share a carpool with (name) each day” to “How likely are you to drive this person to work if you could meet?” In the end, we needed to replace the word carpool but also add an element of convenience to fit the Swedish context. We then sent the survey for back translation. Finally, we had 22 physicians complete a pilot survey but also provide comments on the questions. A few pilot participants commented that it was clear that the survey was not developed in the Swedish context. However, the pilot participants completed the surveys and we examined responses for any missing results or large ranges between answers. All changes were discussed with the research team which included a co-creator (PWC) of the original version of the Attribution Questionnaire and who was involved in this process. Consensus among the research team was needed before any items were modified from their original version. Comments and changes were systematically recorded throughout this process.

For the anger sub-scale, we deleted two items due to semantic equivalence based on advice from the expert committee, cognitive testing and the pilot survey comments. The comments were largely unanimous that the meaning between the three items in the anger scale were not different enough to warrant the three questions and led to participant frustration. For example, angry, irritated and aggravated do not translate into Swedish with enough semantic difference to be meaningful to the respondents. As such, the anger sub-scale was each reduced to two items.

The original responsibility and pity sub-scales each have three items and all of them were preserved in the cultural translation. Semantic-equivalence was needed for all items but no major modifications.

### Sample

We posted the questionnaire to 6,000 Swedish psychiatrists and GPs, representing all of the psychiatrists and a random sample of approximately two thirds of GPs. 1,414 (23.6%) self-completed the questionnaire via post or online. Inclusion criteria were (1) registered practitioner in Sweden and (2) a psychiatrist or GP. We received addresses from the Hälso och sjukvårdens adressregister (Swedish register of health professional addresses). Hälso och sjukvårdens adressregister provided background information such as age, sex and location. Statistics Sweden administered the survey, then returned the anonymised results.

### Statistical analysis

We used Cronbach’s alpha to estimate the instrument reliability with an acceptable level of > 0.70 [26]. We used Structural Equation Modelling (SEM) to test the responsibility model of stigma for the Swedish version of the AQ-27. We used several goodness-of-fit indicators to assess the model and used Schreiber, Nora [27] cut-off criteria: chi-square absolute and predictive fit (nonsignificant *χ*^2^ indicates a good fit), root mean square error of approximation (RMSEA) (RMSEA,0.06 to 0.08 indicates acceptable fit), Tucker-Lewis index (RLI ≥ 0.95), and Comparative fit index (≥ 0.95 for acceptance). We also conducted confirmatory factor analysis (CFA) to confirm that the factors in the Swedish versin loaded into the correct constructs (see Additional file 1: Tables 2 and 3). CFA results confirmed factor loading.

This study was approved by the Stockholm Regional Ethics Board (Dnr 2018/683-31/5).

## Results

Table 1 summarizes the participant characteristics. Gender distribution among participants was relatively similar. Participant’s ages ranged from 25 to 90 (mean = 52.85, SD = 13.23). Most participants worked in the public setting (60%) and had experience completing a disability pension assessment.

### Instrument reliability

Overall, internal consistency reliability for the Attribution Questionnaire-Swedish version (AQ-S) was acceptable (*α* =  0.733). Table 2 provides Cronbach’s alpha coefficients for each factor and compares these results with the Attribution Questionnaire translation of Italian version (AQ-I) [9], the Spanish version (AQ-E) [10].

**Table 2:** Comparison of Cronbach’s alpha coefficients in studies using the Attribution Questionnaire

Factor	Cronbach’s alpha’s coefficient (α)		
	AQ-S (n = 1,414)	AQ-I [9] (n = 214)	AQ-E [10] (n = 439)
Responsibility	0.819	0.615	0.390
Pity	0.633	0.676	0.494
Anger	0.873	0.521	0.577
Help	0.713	0.814	0.766
Overall	0.733	0.818	0.855

Table has been modified from Muñoz, Guillén [10]

AQ-S, Attribution Questionnaire Swedish Version; AQ-I, Attribution Questionnaire Italian Version; AQ-E, Attribution Spanish version

### Responsibility model of stigma

**Fig. 2:**
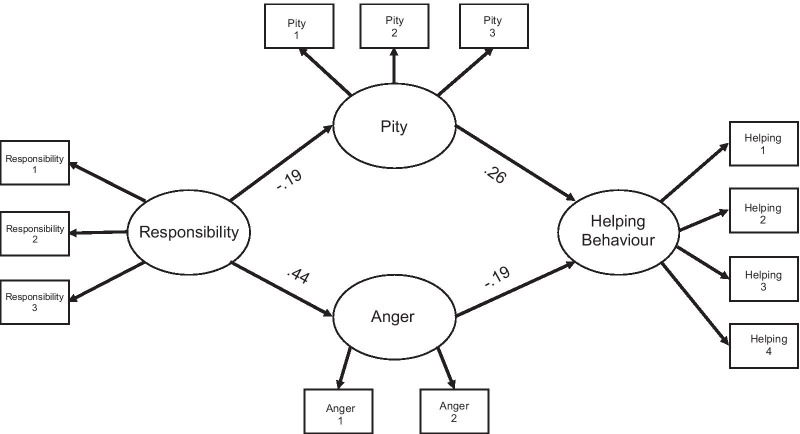
The four-factor measurement of the responsibility model. Note: The rectangles represent observed variables and the ovals represent the unobserved latent variables

The final analytic sample was 1,323. The model has four latent variables: Responsibility (Responsibility 1, Responsibility 2, Responsibility 3), Pity (Pity 1, Pity 2), Anger (Anger 1, Anger 2) and Helping Behaviour (Helping Behaviour 1, Helping Behaviour 2, Helping Behaviour 3, Helping Behaviour 4). Standardized path coefficients are provided on each arrow in Fig. 2. Chi-squared was significant, *χ* ^2^(50) = 465.417, *ρ* < 0.001. The comparative fit index = 0.916 and the RMSEA (0.079) indicate a reasonable fit. The Tucker Lewis index = 0.889 was below the criterion level. Overall, data supports that the model is an acceptable fit.

## Discussion

Reducing stigma towards those with mental illness remains a key priority of many governments [1, 28, 29]. The Swedish Government’s commitment to reduce stigma demonstrates the need for appropriate measurements available in the local language to monitor how attitudes are changing. In this study, we wanted to determine if the American designed Attribution Questionnaire could be culturally translated for Swedish physicians. The Attribution Questionnaire could be a helpful tool for measuring and monitoring stigma in the Swedish setting. Overall, results showed that the questionnaire is suitable for measuring physicians’ attitudes in the Swedish context. However, modifications were necessary to make the questionnaire externally valid in the new setting.

Results from our SEM model are acceptable [27] but the correlations between the latent factors are weaker than previous studies [4, 14, 15]. Similar to Akyurek, Efe [17] we found a negative and weak relationship between the responsibility and pity latent factors compared to other translated versions that found a stronger relationship [14, 15]. However, our sample, comprised only of physicians, differed from the other studies that included mostly students [4, 18] one including medical students [19], or the general population [14, 15]. Physicians, in general, would have more contact with those with mental illness compared to the other studies’ sample populations. As such, the theoretical model may be more applicable to general populations than physicians. Furthermore, since we used a truncated version of the Attribution Questionnaire is difficult to directly compare results with previous version of the questionnaire.

### The importance of the expert committee

The expert committee played a significant role in modifying the questionnaire, much more than the process of back-translation. Previous questionnaire translation emphasises the role of back-translation as best practice for translating questionnaires [30, 31]. However, Epstein, Osborne [24] argue that an expert committee can play more pivotal role when translating across cultures. Our findings support this assertion, indeed, the questionnaire benefited greatly from the use of an expert group. It was the expert committee not the back-translation that identified the semantic issues and found experiential equivalence. Overall, our study supports the inclusion of an expert committee for culturally translating a questionnaire.

### Cultural adaptation and the validity of the questionnaire

Overall, we found that cultural adaptation makes the questionnaire more relevant to the local context. Our results support that questionnaires should be translated for language *and* culture [21–23, 32, 33]. External validation—whether the results can be generalised or useful outside of the research context—is essential. Cultural translation increases the external validity of the questionnaire and ultimately the usefulness of results. Internal consistency—how well a survey measures what you want to measure—is also important and can be impacted by cultural translation. Our results showed that Cronbach’s alpha for some factors, in particular, ‘Responsibility’ were much higher compared to the Italian and Spanish translated versions of the Attribution Questionnaire (Responsibility ***α***: AQ-S = 0.819, AQ-I = 0.615 and AQ-E = 0.390) [14, 15].

### Limitations

This study is part of a broader project on physicians’ attitudes towards disability pension applicants in Sweden. As such, the vignettes used with the survey were changed from the original American vignette of Harry with schizophrenia to Johan/Johanna with depression, alcohol dependence or low back pain. Such changes could explain why the internal consistency varies between the different translated versions. It is difficult to determine if the results from the SEM for the Attribution Questionnaire are not as strong due to the modifications made by the research team or the cultural differences between the contexts or both [15]. Due to feasbility contstraints, our sample included only two thirds of GPs registered in Sweden. A third party—the Hälso och sjukvårdens adressregister—provided the addresses and randomly selected the two thirds before releasing the data for our use. As such, this could impact on the quality of our sample. Survey response rate was low, which could bias the results. Nevertheless, the sample size was bigger than that of many other studies, and concerned two groups of physicians that more frequently deal with disability pension applicants in their work. We also could have included persons with lived experience of mental illness in the expert committee to get their views on the cultural translation. Finally, we were unable run the SEM analysis and the impact on the model of different type of providers e.g. GPs versus psychiatrists.

### Strengths

To the best of our knowledge, only one other study has culturally translated the Attribution Questionnaire [17]. Additionally, compared to the other Attribution Questionnaire translations [14, 15], our sample size is much larger. As such, our results could be more reliable in terms of the SEM and internal consistency analysis. Our results also demonstrate that the Attribution Questionnaire can be modified to suit specific contexts or sub-groups and still remain a meaningful measure of stigma.

### Implications for policy

Given the Swedish Government’s initiative to reduce stigma, we recommend that the AQ-S could be an efficient tool to measure and monitor stigma in the Swedish setting, especially among physicians but also the wider population. In particular, Swedish policy-makers could use the AQ-S to determine whether newly implemented anti-stigma strategies are working. However, the AQ-S should be validated among other sub-groups.

### Implications for practice

Disability pension is not a universal program. Government adjudicators must decide who ‘deserves’ to receive disability pension and who does not. However, these decisions must be based on unbiased information and not discriminatory attitudes. The AQ-S is practical tool to help gauge whether disability pension applicants, regardless of their diagnosis, receive a fair assessment. While this study focuses on this in relation to physicians, the AQ-S could also measure stigma among adjudicators themselves.

### Implications for future research

Further examination of intersectionality—the idea that various forms of social stratification can intersect to create cumulative discrimination or disadvantage [34] in the context of disability pension is vital. While this study explored the intersection of gender and disability, we intend to expand this exploration to include ethnicity and other factors e.g. socioeconomic status in future studies. We need a more nuanced understanding of what factors might lead to positive or negative attitudes among physicians and other policy-makers. The vignettes created in this study could be amended to explore such ‘intersectional stigma’ [35]. A better understanding of intersectional stigma and disability pension would also provide clearer pathways for policy intervention to address any inequalities that may exist.

## Conclusion

Our findings demonstrate that the Swedish version of the Attribution Questionnaire (AQ-S) provides a validated instrument with acceptable psychometric properties for the assessment of stigma among physicians in Sweden.

## Supplementary Information

**Additional file 1:** **Table 1**. Original Attribution Questionnaire. **Table 2**. Rotated factor loadings based on confirmatory factor analysis of the AQ. **Table 3**. Concordance between AQ scales and factor loadings.

## Data Availability

The data are not publicly available due to privacy or ethical restrictions.

## Abbreviations

AQ: Attribution Questionnaire
AQ-I: Attribution Questionnaire Italian Version
AQ-E: Attribution Questionnaire Spanish Version
AQ-S: Attribution Questionnaire Swedish version
CFA: Confirmatory Factor Analysis
GP: General practitioners
RMSEA: Root mean square error of approximation
SEM: Structural Equation Modelling

## Footnotes

1 In survey development, cognitive testing can be used as a method to ensure the quality of the survey items and ensure that the interpretation of the questions is as intended. In this study, we cognitively tested the questionnaire with 3 participants that had medical or mental health backgrounds. The participants were asked to complete the questionnaire prior to meeting. We spent 90 min going through the questionnaire using think-aloud techniques—asking participants to talk through their answers and verbal probing—asking participants what the questions means to them. The interviewer took fieldnotes and documented comments made by the participants. Comments were summarised and then discussed with the project team and also the expert committee.
