# Supplementary material for: Cultural adaptation and validation of the Attribution Questionnaire for stigma towards disability pension applicants for use among psychiatrists and general practitioners in Sweden
Source: BMC Psychol. 2021 Feb 8;9:27. doi: 10.1186/s40359-021-00523-8 (PMC7869470; doi:10.1186/s40359-021-00523-8)
Supplement: Supplementary file 1 — Additional file 1: Table 1. Original Attribution Questionnaire. Table 2. Rotated factor loadings based on confirmatory factor analysis of the AQ. Table 3. Concordance between AQ scales and factor loadings. [file 40359_2021_523_MOESM1_ESM.docx]

**Additional file 1: Table 1. Original Attribution Questionnaire [4]**

| **Personal responsibility beliefs**   1. I would think that it were Harry's own fault that he is in the present condition. (1 = no, not at all; 9 = yes, absolutely so) 2. How controllable, do you think, is the cause of Harry's present condition? (1 = not at all under personal control; 9 = completely under personal control) 3. How responsible, do you think, is Harry for his present condition? (1 = not at all responsible; 9 = very much responsible) |
| --- |
| **Pity**   1. I would feel pity for Harry. (1 = none at all; 9 = very much) 2. How much sympathy would you feel for Harry? (1 = none at all; 9 = very much) 3. How much concern would you feel for Harry? (1 = none at all; 9 = very much) |
| **Anger**   1. I would feel aggravated by Harry. (1 = not at all; 9 = very much) 2. How angry would you feel at Harry? (1 = not at all; 9 = very much) 3. How irritated would you feel by Harry? (1 = not at all; 9 = very much) |
| **Fear**   1. How dangerous would you feel Harry is? (1 = not at all; 9 = very much) 2. I would feel threatened by Harry? (1= no, not at all; 9 = yes, very much) 3. How scared of Harry would you feel? (1 = not at all; 9 = very much) 4. How frightened of Harry would you feel? (1 = not at all; 9 = very much) |
| **Helping**   1. If I were an employer, I would interview Harry for a job. (1 = not likely; 9 = very likely) 2. I would share a carpool with Harry each day. (1 = not likely; 9 = very likely) 3. How certain would you feel that you would help Harry? (1 = not at all certain; 9 = absolutely certain) 4. If I were a landlord, I probably would rent an apartment to Harry. (1 = not likely; 9 = very likely) |
| **Coercion-Segregation**   1. I think Harry poses a risk to his neighbors unless he is hospitalized. (1 = not at all; 9 = very much) 2. I think it would be best for Harry's community if he were put away in a psychiatric hospital. (1 = not at all; 9 = very much) 3. How much do you think an asylum, where Harry can be kept away from his neighbors, is best? (1 = not at all; 9 = very much) 4. If I were in charge of Harry's treatment, I would force him to live in a group home. (1 = not at all; 9 = very much) |

**Additional file 1: Table 2. Rotated factor loadings based on confirmatory factor analysis of the AQ**

Bold values indicate best loading score for each identified factor. AQ – Attribution Questionnaire

| **Variable** | **Factor 1** | **Factor 2** | **Factor 3** | **Factor 4** |
| --- | --- | --- | --- | --- |
| F11 | **0.8225** | -0.1153 | 0.2258 | -0.0162 |
| F12 | **0.8188** | 0.0508 | 0.1661 | -0.0653 |
| F13 | **0.8530** | -0.0123 | 0.1374 | -0.0459 |
| F14 | -0.1116 | 0.0957 | 0.2035 | **0.7301** |
| F15 | -0.1854 | 0.1333 | -0.2203 | **0.7199** |
| F16 | 0.0844 | -0.0033 | 0.0351 | **0.8032** |
| F17 | 0.1472 | -0.0451 | **0.9281** | 0.0084 |
| F18 | 0.2145 | -0.0973 | **0.9011** | 0.0135 |
| F22 | 0.0264 | **0.5866** | -0.0558 | -0.1125 |
| F23 | 0.0264 | **0.7856** | -0.1046 | 0.0847 |
| F24 | 0.0766 | **0.7882** | -0.0968 | 0.2141 |
| F25 | -0.2322 | **0.7322** | -0.0218 | -0.0599 |

**Additional file 1: Table 3. Concordance between AQ scales and factor loadings**

| **Factor Number** | **Scale Name** | **AQ Scale Items** |
| --- | --- | --- |
| Factor 1 | Responsibility | F11-F13 |
| Factor 2 | Helping | F14-F16 |
| Factor 3 | Anger | F17 & F18 |
| Factor 4 | Pity | F22-F25 |

AQ – Attribution Questionnaire
